# Powdery Mildew Resistance Genes in Single-Plant Progenies Derived from Accessions of a Winter Barley Core Collection

**DOI:** 10.3390/plants10101988

**Published:** 2021-09-23

**Authors:** Antonín Dreiseitl, Zdeněk Nesvadba

**Affiliations:** 1 Department of Integrated Plant Protection, Agrotest Fyto Ltd., Havlíčkova 2787, CZ-767 01 Kroměříž, Czech Republic; 2 Gene Bank, Crop Research Institute, Drnovská 507, CZ-161 06 Praha 6-Ruzyně, Czech Republic; nesvadba@vurv.cz

**Keywords:** *Blumeria graminis* f. sp. *hordei*, gene bank, *Hordeum vulgare*, isolates of the pathogen, infection response arrays, resistance gene postulation, winter barley core collection

## Abstract

The main problems of crop gene banks comprise heterogeneity of accessions, resulting from mechanical admixtures or out-crossing during their multiplication, and especially the mislabeling of accessions. These discrepancies can adversely affect the results of many expensive research and breeding projects that are based on the use of gene bank resources. To tackle these problems, 860 single-plant progenies (SPPs) of 172 accessions of the Czech winter barley core collection were grown and tested with a set of 53 isolates representing the global virulence/avirulence diversity of powdery mildew. Seventy-one resistance phenotypes encompassed the diversity of known specific resistances and their combinations. Based on testing groups of five SPPs, 94 accessions had one phenotype found in all five SPPs (homogeneous accessions), whereas in 78 accessions (45.3%) more than one phenotype was identified (heterogeneous accessions). In three varieties, specific resistances against the whole set of isolates were detected, but due to high adaptability of the pathogen, they are not recommended for breeding resistant cultivars. Selected SPPs were integrated in the gene bank and are now a reliable source of genotypically pure seed with defined powdery mildew resistance genes that can be used by breeders and researchers. The results obtained can be used to verify authenticity of accession genotype and pedigree, particularly for older varieties for which no other original criteria are available.

## 1. Introduction

Barley (*Hordeum vulgare* L.) is an important cereal crop used mainly as human food, feed for domestic animals and for malt products. Powdery mildew, caused by the fungus *Blumeria graminis* (D.C.) Golovin ex Speer, f. sp. *hordei* Em. Marchal (*Bgh*), is a worldwide disease that can cause frequent epidemics on barley particularly in Central [1] and Northwest Europe [2,3]. To reduce the disease and its effect on yield and quality, genetic resistance is an efficient and environmentally acceptable way [4,5] and has played an essential part in disease management for about seven decades [6].

Breeding barley resistant to powdery mildew began in Europe in the interwar period [7]. The first resistance genes used in commercial varieties included *Mlg* in spring and *Mlh* and *Mlra* in winter cultivars. The first cultivar with a gene located at the Mla locus was Vogelsanger Gold (*Mla6*) derived from wild barley (*Hordeum vulgare* subsp. *spontaneum*) and many subsequent cultivars were bred with other new resistances, mainly alleles of Mla locus [6,8]. Later, it was found that even European landraces carry some specific resistance genes completely ineffective in the field [9].

*Bgh* is a highly adaptable pathogen, and its Central European population is extremely diverse because it reflects a great diversity of resistances used in breeding programmes [10]. More than 70 resistance genes [9] have been described and most of them are present in cultivars either singly or in various combinations, whereas cultivars without a resistance gene make up only a small proportion.

Recently different resistance genes at Mla locus and *mlo* gene were molecularly isolated [11]. Isolates selected from the global population of the pathogen can reveal the detailed genotypic diversity of the host and this knowledge was used to characterize cultivars including verification of their homogeneity and authenticity of their genotype and pedigree [12,13]. This is particularly needed for older accessions for which there is little to no genomic information available, like molecular marker profiles generated on the original seed stocks. Hence, molecular technology alone cannot be used for confirming their identity without additional testing. If new methods are used for characterizing accessions of old varieties without confirmation their seed authenticity and purity, it can lead to a series of wrong results, loss of the original cultivar identity and decreased genetic diversity in gene banks.

This contribution describes the identification of the powdery mildew resistance genes in single-plant progenies (SPPs) of accessions of the core collection (CC) of the Czech winter barley gene bank. Results from a similar project have already been reported [8], but more than 85% of the accessions showed heterogeneity in their resistance and identification of resistances was limited. Therefore, the aims of this study were i) to grow a technically-manageable group of homogeneous samples (lines) in order to record the most frequent genotypes present in each accession; ii) to identify resistance genes in each of them, and iii) to evaluate their authenticity. Together, this aims at providing breeders and researchers with a large set of single-line barley germplasm with well-defined powdery mildew resistance genes.

## 2. Results

All 860 SPPs derived from 172 gene bank accessions of winter barley were characterized by homogeneous infection response arrays (IRAs) confirming the genotypic uniformity of these single-line samples. From the tests 71 IRAs were recorded that represented the phenotypes of specific resistance genes and their combinations (Table 1). Based on testing groups of five SPPs, 94 accessions had one identical phenotype found in all SPPs (homogeneous accessions) whereas, 78 accessions (45.3%) were heterogeneous in which two, three, four and five genotypes were found in 46, 19, 10 and 3 accessions, respectively. Based on the name of each accession that was tested and IRAs, 298 variants were found. The resistance genes of all 860 SPPs are presented in Table S1.

Twenty-three known *Ml* resistance genes were identified in 761 SPPs (Table 2), the most frequent being *aLo*, *ra*, *a8*, *h* and *Ch* in 237, 220, 154, 139 and 112 SPPs, respectively. Some genes, especially *a8*, *Ch*, Dr2 and *ra*, may also be present in other varieties because their phenotype is masked by commonly occurring resistance genes such as *g*, *a6*, *a7*, *a12* or *a13*. Conversely, the least frequent were *He2* found in two SPPs, *Ln* in six SPPs of two accessions, *a3* in seven SPPs of three accessions, and *at*, *Dt6*, *St*, and *Wo* each present in all five SPPs of Local (Merkez-Kaza), Duet, Traminer and Wong, respectively.

In total, 1363 known genes were recorded in 761 SPPs with identified resistances equivalent to an average of 1.79 specific genes per SPP. In 525 of them (69.0%) genes were located at the Mla locus. IRAs of 28 SPPs did not correspond with the IRAs of the reported resistances and were, therefore, designated as unknown (*u*), 20 SPPs relating mostly to Bonita, Marconee and Mc Nair 601 were resistant to all isolates (*e*) and conversely in 51 SPPs no resistance gene was found when in 10 varieties (Bankuti 14, Bordia, Dagestanskij-Samuricum 293, Krakovski, Krasnodarskij 2929, Krusevacki, Nakaizumi Zairai, Opolski 152, Stupicky dvourady and Zalarinec) susceptible SPPs with no detected resistance gene (designated ‘none’ in Table S1) predominated.

## 3. Discussion

The resistance of 19 of the 172 accessions is presented in the catalogue of European varieties [6], but only two of which (Frolic and Perga) had identical resistance to those reported here and only *Mla7* was found in Marinka whereas *Mla7* and *Mlg* are listed in the catalogue for this cultivar.

In nine accessions (Breustedts Atlas, Breustedts Schladener I, Carsten Zweizeilige, Dana, Eckendorfer Glatta, Engelens Dea, Fimbull II, Hauters Wintergerste and Strengs Dura), there were more resistance genes in addition to those published and reflects the high resolution of the large set of carefully selected isolates used herein. An example of the refinement of earlier results is *MlaLo*, the most frequent gene found here, including the first five out of nine accessions mentioned above. This gene was not known until recently. Formerly [14,15], RT0 was found in some varieties after testing with the Japanese isolate Race I [16], which is typical for *Mla8* often present in spring varieties [17]. The same response was subsequently detected and is typical for the newly-discovered gene *MlaLo* [18], which is allelic to *Mla8* [19] and, in contrast to *Mla8*, is characteristic of winter barley.

In the previous paper [8], approximately 50 plants grown from seeds of an accession stored in the gene bank were used for resistance tests and heterogeneity was found in 147 out of 172 accessions of the core collection. In this paper, only five SPPs were tested from each accession, among which different genotypes in 78 accessions (45.3%) were found. In 77 of them, heterogeneity had also been uncovered previously, while in Alterna, heterogeneity was revealed for the first time and was caused by the presence of two genotypes with similar IRAs conditioned by the genes *Mla8* and *Ml(Ch)*.

The number of heterogeneous accessions in the collection is high, but not all heterogeneity could be detected for several reasons [8], including testing only a limited number of SPPs. Heterogeneity of gene bank accessions can have several causes relating to breeding methods or collecting landraces, out-crossing and mechanical admixtures. Cv. Will is an example of heterogeneity resulting from a breeding method without selecting for mildew resistance in which two genotypes (*Mla7* and *Mla7*, *Mlh*) were recorded. *Mla7*, which was present in all five SPPs confirms that they originated from an identical crossing programme and that the presence or absence of *Mlh* has resulted in the existence of a second line. A similar and previously mentioned case could be Marinka which possesses *Mla7* and *Mlg* according to the catalogue [6], whereas, a line possessing only *Mla7* was identified herein once again [8].

The best example of the absence of resistance gene selection in a population derived from an identical origin is Ragusa 34 ¬-40. Among five SPPs, five genotypes contained five *Ml* genes in different combinations (*a8*; *a8, h*; *a8, h, ra*; *Ch, h, ra* and *Ch, Dr2, ra*). Since such a number of genes can result in more different genotypes, many SPPs of this accession should be tested to uncover all existing combinations. Conversely Ventitre is an example of an accession that is composed of genotypes that could not have an identical source. In two SPPs there was a genotype with three *Ml* genes (*aLo, ra, Ru2*), whereas, in three other lines only one different gene (*Mla8*) was found. These two genotypes have no resistances in common and the accession is a mixture of different varieties.

Beside detection of heterogeneity the results enable to uncover mislabelled accessions. In seven accessions as well as in two other varieties published elsewhere, the resistance genes differed from those listed in the catalogue (Table 3). For example, although *Ml(Bw)* has been recorded in Angela [20], in our tests Angela has three different *Ml* genes (*h, (Lu)* and *ra*) and clearly demonstrates the difference between “our“ accession and that used previously. In the catalogue, Borwina has *Mla6* although at the time of its registration in the Czech Republic (1983) this variety had a resistance phenotype that differed from all other winter varieties. Therefore, it was considered a new gene and designated according to this variety *Ml(Bw)* [21]. This resistance was soon discovered in other Central European winter varieties [22], and subsequently in many Chinese barleys [23]. Later it was found to be identical to the *Ml(Ru2)* present in one (P15) of the near-isogenic lines of the spring variety Pallas [24,25]. *Ml(Ru2)* has so far only been found in winter varieties (except P15) and in our study in 73 SPPs. Thus, in addition to the *Ml* genes *aLo, ra* and *h, Ml(Ru2)* can be included among the typical resistances of winter barley. Two genotypes were found in the group of Borwina SPPs, both carry *Ml(Ru2)*, but differ in having five other resistance genes.

Old varieties are predominant in CCs and have undergone several propagation cycles, each of which might result in possible errors. Mislabeling and contamination of accessions could occur mainly at the time when the rules for working in the nascent gene banks were not sufficiently specified and technical equipment was not able to maintain seed with a high degree of purity. Therefore, the differences found in the resistance of nine of the varieties (Table 3) can be explained by the probable mislabeling of the accessions. However, an identical commercial name used for different varieties cannot be ruled out as with two spring varieties Opal [6,22], Freya in which the spring type has *Mlg* [26] and winter type has *Mla6* (this contribution) and Zenit in which both spring and winter types have *Mla13* [8,22].

In this report three varieties with resistance genes effective against all the pathogen isolates used were found. In other tests many varieties with a similar type of resistance to different sets of pathogen isolates have been identified. This indicates a lack or less intensive directional selection of the pathogen due to the absence or low proportion of barley varieties with appropriate resistances rather than the effectiveness and especially the durability of these resistances. Many of varieties with reported effective resistances, including Bonita studied here, were used in a set of 95 differential varieties in the current (2021) population study, and although only 72 isolates were obtained, new and rare virulences to several these resistances were detected [27].

The rapid adaptation of the pathogen excludes the successful use of specific resistance genes even though they appear to be fully effective, because specific resistance genes are quickly overcome and for this reason their use in breeding barley for resistance against powdery mildew is no longer recommended [9,28]. In winter barley, it is advisable to focus on the accumulation of minor genes that are predominantly non-specific [29,30,31]. An alternative strategy could be to use the resistance of *Hordeum bulbosum*, the only representative of the secondary genepool of cultivated barley [32], although the resistance of the three so far derived genes [33,34,35] to pathogen adaptation has not been sufficiently tested.

## 4. Materials and Methods

The following parts, especially 4.2. and 4.3. are similar to those previously described [8].

### 4.1. Plant Material and Pathogen Isolates

A set of individually sown plants from each of 172 accessions of the CC of the Czech gene bank of winter barley originating from 35 countries were grown in rows in the field and five single-plants of each accession were harvested and investigated. For resistance tests, 53 selected reference isolates of *Bgh* were used, which had been collected in 11 countries in all nonpolar continents over a period of 63 years (1953–2016) and represents the global virulence/avirulence diversity of the pathogen [36].

### 4.2. Testing Procedure

About 20 seeds of an ear of each SPP was sown in a pot (80 mm diameter) and grown in a mildew-proof greenhouse under natural daylight. The primary leaves were excised when the second leaves were emerging, and segments 20 mm long were cut from the middle part of healthy fully-expanded leaves. One leaf segment of each SPP was placed on the surface of water agar in a 150 mm Petri dish and each dish was separately inoculated with the pathogen isolates in a concentration of ca. 10 conidia mm^−2^.

### 4.3. Evaluation

**Figure 1:**
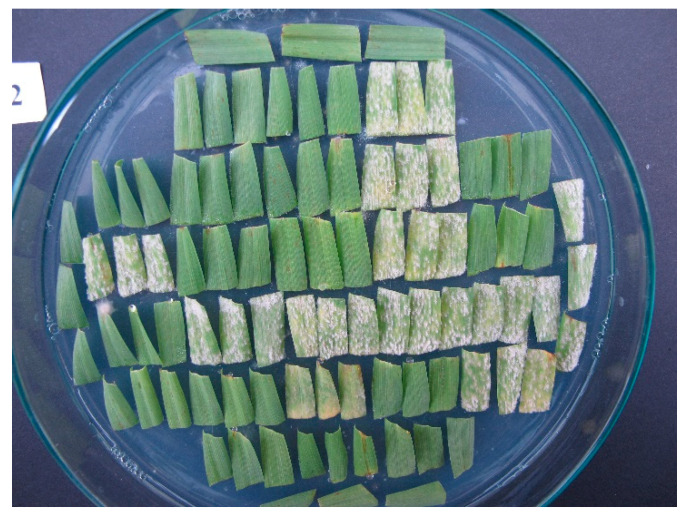
Infection responses (IRs) produced by a *Blumeria graminis* f. sp. *hordei* isolate on 30 barley genotypes each represented with a triplet of leaf segments seven days after inoculation; three IRs are shown here, IR 0—full resistance (green leaf segments), IR 4—full susceptibility (leaf segments with colonies of white conidia of the pathogen) and IR 2—moderate resistance (colonies of grey mycelium surrounded with chlorotic/necrotic spots) on a triplet in the middle of the last long row. This figure is a demonstration picture from other tests.

Seven days after inoculation, infection responses (IR = phenotype of SPP × isolate interaction) on the middle part of the adaxial side of leaf segments were scored on a scale 0–4, where 0 = no mycelium and sporulation, and 4 = strong mycelial growth and sporulation (Figure 1) [14]; IRs 3, 3–4 and 4 were considered susceptible. Each SPP was tested with a minimum of one replication but most SPPs were included in two or more replications. A set of 53 IRs provided an IRA for each SPP. Based on the gene-for-gene model [37] the resistance genes in SPPs were postulated by comparing the IRAs with previously determined IRAs of standard barley genotypes possessing known resistance genes.

## 5. Conclusions

- The main problems of gene banks include mislabeling of accessions, heterogeneity resulting from mechanical admixtures or out-crossing during their multiplication and low germination. All these problems can adversely affect the results of research and breeding projects that are based on the use of gene bank resources. Therefore, the highest priority of plant gene banks curators must be to provide breeders and researchers with authentic seed of original genotypes.
- From all 172 accessions of the given CC, 860 homogeneous lines (SPPs) were created and their genetic basis of resistance to powdery mildew was studied.
- More than one genotype was identified among SPPs of 78 accessions (=45.3% heterogeneous accessions).
- Only 21 accessions (12.2%) were found to have data previously published on their resistance and the resistance genes identified here often differed.
- Selected lines (SPPs) of the CC accessions were multiplied in the field and stored in the gene bank, and are freely available for the use of breeders and researchers [38].
- Seeds of many accessions were requested from other gene banks and from each of them SPPs were also grown. These are studied in a similar way to assess their homogeneity and authenticity. Accessions whose authenticity is questionable will be replaced with genuine ones.
- Rules for replacing accessions with questionable identity and using genotypes derived from heterogeneous accessions require an international agreement.

## Figures and Tables

**Table 1:** Infection response arrays produced by 11 *Blumeria graminis* f. sp. *hordei* isolates on 71 barley genotypes and their powdery mildew resistance genes.

*Ml* Gene(s)	Race I	J-462	EA30	PF512	C-132	3-33	65	GH	54	Z-6	E-6
none	4 ^1^	4	4	4	4	4	4	4	4	4	4
*a3*	0	4	0	0	4	0	0	0	0	4	0
*a6*	0	4	2	4	4	0	0	0	0	4	0
*a6, aLo*	0	0	2	4	4	0	0	0	0	4	0
*a6, Dt6, g, h*	0	4	0	4	1–2	0	0	0	0	2	0
*a6, h*	0	4	2	4	1–2	0	0	0	0	4	0
*a6, h, Lu, ra, Ru2*	0	4	0–1	2–3	1–2	0	0	0	0	1–2	0
*a6, h, ra*	0	4	0–1	4	1–2	0	0	0	0	4	0
*a6, IM9*	0	0	0	4	0	0	0	0	0	0	0
*a6, ra*	0	4	0–1	4	4	0	0	0	0	4	0
*a7*	0	0	1–2	4	4	0	0	1–2	1–2	4	4
*a7, h*	0	0	1–2	4	1–2	0	0	1–2	1–2	4	4
*a8*	0	4	4	4	4	4	4	4	4	4	4
*a8, Dr2*	0	4	4	4	4	4	4	2–3	4	4	4
*a8, Dr2, ra*	0	4	0–1	4	4	4	0–1	2–3	4	4	4
*a8, Dr2, ra, VIR*	0	4	0–1	1	4	4	0–1	2–3	4	4	4
*a8, h*	0	4	4	4	1–2	1–2	4	1–2	1–2	4	4
*a8, h, Ln, ra*	0	4	0–1	0–1	1–2	1–2	0–1	1–2	1–2	0–1	0–1
*a8, h, Lu, Ru2*	0	4	4	2–3	1–2	1–2	1–2	1–2	1–2	1–2	4
*a8, h, ra*	0	4	0–1	4	1–2	1–2	0–1	1–2	1–2	4	4
*a8, h, ra, Ru2*	0	4	0–1	2–3	1–2	1–2	0–1	1–2	1–2	2–3	4
*a8, He2*	0	4	4	4	4	4	4	4	2–3	4	4
*a8, Lu, ra*	0	4	0–1	4	4	4	0–1	1–2	4	1–2	4
*a8, ra*	0	4	0–1	4	4	4	0–1	4	4	4	4
*a8, Ru2*	0	4	4	2–3	4	4	2–3	2–3	2–3	2–3	4
*a8, VIR*	0	4	4	1	4	4	4	4	4	4	4
*a12*	1	4	4	4	4	1	1	1	1	4	4
*a12, aLo, g, Lu*	0	0	0	4	4	0	0	1	1	1–2	4
*a12, g*	0	4	0	4	4	0	0	1	1	4	4
*a13*	0	0	0	0	4	0	0	0	0	0	4
*aLo*	0	0	4	4	4	4	4	4	4	4	4
*aLo, Dr2*	0	0	4	4	4	4	4	2	4	4	4
*aLo, Dr2, ra*	0	0	0–1	4	4	4	0–1	2	4	4	4
*aLo, g*	0	0	0	4	4	0	0	4	4	4	4
*aLo, h*	0	0	4	4	1–2	1–2	4	1–2	1–2	4	4
*aLo, h, Lu, ra*	0	0	0–1	4	1–2	1–2	0–1	1–2	1–2	1–2	4
*aLo, h, Lu, ra, Ru2*	0	0	0–1	2–3	1–2	1–2	0–1	1–2	1–2	1–2	4
*aLo, h, ra*	0	0	0–1	4	1–2	1–2	0–1	1–2	1–2	4	4
*aLo, Lu*	0	0	4	4	4	4	1–2	1–2	4	1–2	4
*aLo, Lu, ra*	0	0	0–1	4	4	4	0–1	1–2	4	1–2	4
*aLo, Lu, ra, Ru2*	0	0	0–1	2–3	4	4	0–1	1–2	2–3	1–2	4
*aLo, Lu, Ru2*	0	0	4	2–3	4	4	1–2	1–2	2–3	1–2	4
*aLo, ra*	0	0	0–1	4	4	4	0–1	4	4	4	4
*aLo, ra, Ru2*	0	0	0–1	2–3	4	4	0–1	4	2–3	2–3	4
*aLo, Ru2*	0	0	4	2–3	4	4	2–3	2–3	2–3	2–3	4
*aLo, VIR*	0	0	4	1	4	4	4	4	4	4	4
*at, h*	2	4	2	2	1–2	2	2	1–2	1–2	1–2	2
*Ch*	2	4	4	4	4	4	4	4	4	4	4
*Ch, Dr2*	2	4	4	4	4	4	4	2	4	4	4
*Ch, Dr2, Lu, Ru2*	2	4	4	2–3	4	4	1–2	2	2–3	1–2	4
*Ch, Dr2, ra*	2	4	0–1	4	4	4	0–1	2	4	4	4
*Ch, Dr2, ra, VIR*	2	4	0–1	1	4	4	0–1	2	4	4	4
*Ch, h, ra*	2	4	0–1	4	1–2	1–2	0–1	1–2	1–2	4	4
*Ch, Lu, ra*	2	4	0–1	4	4	4	0–1	1–2	4	1–2	4
*Ch, ra*	2	4	0–1	4	4	4	0–1	4	4	4	4
*Ch, ra, VIR*	2	4	0–1	1	4	4	0–1	4	4	4	4
*Ch, Ru2*	2	4	4	2–3	4	4	2–3	2–3	2–3	2–3	4
*Dr2, ra*	4	4	0–1	4	4	4	0–1	2	4	4	4
*Dr2, ra, VIR*	4	4	0–1	1	4	4	0–1	2	4	4	4
*g*	0	4	0	4	4	0	0	4	4	4	4
*g, Ln*	0	4	0	0–1	4	0	0	4	0–1	0–1	0–1
*h*	4	4	4	4	1–2	1–2	4	1–2	1–2	4	4
*h, Lu, ra*	4	4	0–1	4	1–2	1–2	0–1	1–2	1–2	1–2	4
*h, ra*	4	4	0–1	4	1–2	1–2	0–1	1–2	1–2	4	4
*IM9, St*	0	0	0	0	0	0	0	0	0	0	4
*La, ra*	4	4	0–1	4	4	2–3	0–1	2–3	4	4	4
*Lu, Ru2*	4	4	4	2–3	4	4	1–2	1–2	2–3	1–2	4
*ra*	4	4	0–1	4	4	4	0–1	4	4	4	4
*Ru2*	4	4	4	2–3	4	4	2–3	2–3	2–3	2–3	4
*VIR*	4	4	4	1	3	4	4	3	3	4	4
*Wo*	(2) ^2^	(3)	(3)	(3)	(3)	(3)	(3)	(3)	(3)	(3)	(3)

^1^ Phenotypes (infection responses) of host-pathogen interactions evaluated according to Torp et al. [14], where 0 = resistant and 4 = susceptible. ^2^ Parentheses indicate smaller number of colonies.

**Table 2:** Number of specific resistance genes found in 860 single-plant progenies derived from 172 winter barley gene bank accessions.

*Ml* Genes	Number	*Ml* Genes	Number
*a3*	7	*IM9*	10
*a6*	72	*La*	9
*a7*	25	*Ln*	6
*a8*	154	*Lu*	92
*a12*	14	*ra*	220
*a13*	21	*Ru2*	73
*aLo*	237	*St*	5
*at*	5	*VIR*	23
*Ch*	112	*Wo*	5
*Dr2*	89	Sum	1363
*Dt6*	5		
*g*	38	Effective (*e*)	20
*h*	139	Unknown (*u*)	28
*He2*	2	*none*	51

**Table 3:** Powdery mildew resistance genes found in identically designated winter barley accessions (discrepancies probably resulted from mislabeling of varieties).

Accession	*Ml* Resistance Gene(s)	
	Present Study	Previous Studies
Angela	*h*, *Lu, ra*	*Bw* (=*Ru2*), *ra* ^1^
Borwina	*a8*, *h, ra, Ru2*	*a6* ^2^
Borwina	*aLo*, *Lu*, *Ru2*	
Capri	*aLo*, *Lu*, *Ru2*	*g* ^2^
Erfa	*aLo*, *Lu*	*h*, *u*^2^
Jutta	a8	*a12*, *g*^2^
Leon	*aLo*, *Dr2*, *ra*	*none* ^2^
Leon	*a8*	
Leon	*none*	
Nelly	*a13*	*a7*, *Ab*^1^
Pamina	*aLo*, *Lu*	*a9*, *g*^2^
Pamina	*a6*, *ra*	
Vogelsanger Gold	*a8*	*a6*, *h*, *ra*^2^

^1^ Anonymous [20]. ^2^ Brown and Jørgensen [6].

## Data Availability

All relevant data are involved in the article and supplementary Table S1.

## Supplementary Materials

The following are available online at https://www.mdpi.com/article/10.3390/plants10101988/s1, Table S1. Eight hundred and sixty single-plant progenies of 172 winter barley gene bank accessions, their country of origin and postulated *Ml* resistance genes against powdery mildew.
