# Supplementary material for: Powdery Mildew Resistance Genes in Single-Plant Progenies Derived from Accessions of a Winter Barley Core Collection
Source: Plants (Basel). 2021 Sep 23;10(10):1988. doi: 10.3390/plants10101988 (PMC8537652; doi:10.3390/plants10101988)
Supplement: Supplementary file 1 [file plants-10-01988-s001.zip › plants-1338265-supplementary.pdf]

Table S1. Eight hundred and sixty single plant progenies of 172 winter barley gene bank accessions, their country of origin and postulated *Ml* resistance genes against powdery mildew.

Accession	No.	Country[1]	Ml gene(s)
Ager	1	FRA	*a8, h*
Ager	2		*aLo, ra*
Ager	3		*a8, Dr2, ra*
Ager	4		*aLo*
Ager	5		*aLo*
Agrilo	1	DEU	*Ch, Dr2, ra*
Agrilo	2		*Ch, Dr2, ra*
Agrilo	3		*Ch, Dr2, ra*
Agrilo	4		*Ch, Dr2, ra*
Agrilo	5		*Ch, Dr2, ra*
Aizn Coiled Necn	1	JPN	*a6, h, ra*
Aizn Coiled Necn	2		*a8, He2*
Aizn Coiled Necn	3		*a6, h*
Aizn Coiled Necn	4		*u*
Aizn Coiled Necn	5		*u*
Alaska	1	USA	*aLo*
Alaska	2		*aLo, h*
Alaska	3		*aLo*
Alaska	4		*aLo*
Alaska	5		*aLo*
Alissa	1	DEU	*a6, h, Lu, ra, Ru2*
Alissa	2		*a6, h, Lu, ra, Ru2*
Alissa	3		*a6, h, Lu, ra, Ru2*
Alissa	4		*a6, h, Lu, ra, Ru2*
Alissa	5		*a6, h, Lu, ra, Ru2*
Alterna	1	DDR	*a8*
Alterna	2		*a8*
Alterna	3		*Ch*
Alterna	4		*a8*
Alterna	5		*a8*
Angela	1	FRA	*h, Lu, ra*
Angela	2		*h, Lu, ra*
Angela	3		*h, Lu, ra*
Angela	4		*h, Lu, ra*
Angela	5		*h, Lu, ra*
Anson	1	GBR	*a8*
Anson	2		*a8*
Anson	3		*a8*
Anson	4		*a8*
Anson	5		*a8*
Antoninski	1	POL	*Ch*
Antoninski	2		*Ch*
Antoninski	3		*Ch*
Antoninski	4		*Ch*
Antoninski	5		*aLo*
Argovia	1	CHE	*a6, h, ra*
Argovia	2		*a6, h, ra*

Argovia	3		*a6, h, ra*
Argovia	4		*a6, h, ra*
Argovia	5		*a6, h, ra*
Aviron	1	DEU	*aLo, Dr2, ra*
Aviron	2		*aLo, Dr2, ra*
Aviron	3		*aLo, ra*
Aviron	4		*aLo, ra*
Aviron	5		*aLo, ra*
Babylone	1	FRA	*a6*
Babylone	2		*a6*
Babylone	3		*a6*
Babylone	4		*a6*
Babylone	5		*a6*
Bahadar	1	ETH	*aLo, Dr2*
Bahadar	2		*aLo, Dr2*
Bahadar	3		*aLo*
Bahadar	4		*u*
Bahadar	5		*aLo, Dr2*
Bankuti 14	1	HUN	*none*
Bankuti 14	2		*none*
Bankuti 14	3		*a8*
Bankuti 14	4		*aLo*
Bankuti 14	5		*none*
Beloruskij	1	SUN	*aLo*
Beloruskij	2		*aLo*
Beloruskij	3		*aLo*
Beloruskij	4		*aLo*
Beloruskij	5		*aLo*
Boehmerwaelder	1	DEU	*a8, Dr2*
Boehmerwaelder	2		*aLo*
Boehmerwaelder	3		*aLo*
Boehmerwaelder	4		*aLo, h*
Boehmerwaelder	5		*aLo*
Bonanza	1	CAN	*u*
Bonanza	2		*u*
Bonanza	3		*u*
Bonanza	4		*u*
Bonanza	5		*u*
Bonita	1	AUT	*h, ra*
Bonita	2		*e*
Bonita	3		*e*
Bonita	4		*e*
Bonita	5		*e*
Bordia	1	BEL	*Ch*
Bordia	2		*none*
Bordia	3		*none*
Bordia	4		*none*
Bordia	5		*none*
Borwina	1	DDR	*a8, h, ra, Ru2*

Borwina	2		*a8, h, ra, Ru2*
Borwina	3		*aLo, Lu, Ru2*
Borwina	4		*aLo, Lu, Ru2*
Borwina	5		*aLo, Lu, Ru2*
Breustedts Atlas	1	DEU	*aLo*
Breustedts Atlas	2		*aLo*
Breustedts Atlas	3		*aLo*
Breustedts Atlas	4		*aLo*
Breustedts Atlas	5		*aLo*
Breustedts Schladener	1	DEU	*aLo*
Breustedts Schladener	2		*aLo*
Breustedts Schladener	3		*aLo*
Breustedts Schladener	4		*aLo*
Breustedts Schladener	5		*aLo*
Brucker Vierzeilige No. 4	1	AUT	*aLo, Dr2, ra*
Brucker Vierzeilige No. 4	2		*aLo*
Brucker Vierzeilige No. 4	3		*a8, ra*
Brucker Vierzeilige No. 4	4		*a8, h, ra*
Brucker Vierzeilige No. 4	5		*a8, h, ra*
Brucker Zweizeilige No.34	1	AUT	*a8*
Brucker Zweizeilige No.34	2		*a8*
Brucker Zweizeilige No.34	3		*a8, He2*
Brucker Zweizeilige No.34	4		*a8*
Brucker Zweizeilige No.34	5		*a8*
Camera	1	GBR	*a7, h*
Camera	2		*a7, h*
Camera	4		*a7, h*
Camera	5		*a7, h*
Camera	3		*a7, h*
Capri	1	BEL	*aLo, Lu, Ru2*
Capri	2		*aLo, Lu, Ru2*
Capri	3		*aLo, Lu, Ru2*
Capri	4		*aLo, Lu, Ru2*
Capri	5		*aLo, Lu, Ru2*
Carola	1	DEU	*a6, IM9*
Carola	2		*a6, IM9*
Carola	3		*a6, IM9*
Carola	4		*a6, IM9*
Carola	5		*a6, IM9*
Carstens Zweizeilige	1	DEU	*aLo*
Carstens Zweizeilige	2		*aLo*
Carstens Zweizeilige	3		*aLo*
Carstens Zweizeilige	4		*aLo*
Carstens Zweizeilige	5		*aLo*
Carstenuv dvourady	1	DEU	*aLo*
Carstenuv dvourady	2		*aLo*
Carstenuv dvourady	3		*aLo*
Carstenuv dvourady	4		*aLo*
Carstenuv dvourady	5		*aLo*

Cenad 450	1	ROU	*aLo*
Cenad 450	2		*aLo*
Cenad 450	3		*aLo*
Cenad 450	4		*aLo*
Cenad 450	5		*aLo*
Cenader Sechszeilige Typ B	1	DDR	*Ch*
Cenader Sechszeilige Typ B	2		*Ch*
Cenader Sechszeilige Typ B	3		*Ch*
Cenader Sechszeilige Typ B	4		*Ch*
Cenader Sechszeilige Typ B	5		*Ch*
Cirpan 5652	1	BGR	*a8*
Cirpan 5652	2		*Ch*
Cirpan 5652	3		*a8*
Cirpan 5652	4		*none*
Cirpan 5652	5		*a8*
Clerix	1	FRA	*ra*
Clerix	2		*ra*
Clerix	3		*ra*
Clerix	4		*ra*
Clerix	5		*ra*
Condorcor	1	DZA	*a8*
Condorcor	2		*a8*
Condorcor	3		*a8*
Condorcor	4		*a8*
Condorcor	5		*a8*
Cyklon	1	SUN	*a6*
Cyklon	2		*a6*
Cyklon	3		*a6, ra*
Cyklon	4		*a6, aLo*
Cyklon	5		*a6, ra*
Dagestanskij (Samuricum 293)	1	AZE	*none*
Dagestanskij (Samuricum 293)	2		*none*
Dagestanskij (Samuricum 293)	3		*aLo*
Dagestanskij (Samuricum 293)	4		*aLo*
Dagestanskij (Samuricum 293)	5		*none*
Dana	1	ROU	*aLo*
Dana	2		*aLo, Dr2, ra*
Dana	3		*aLo, Dr2, ra*
Dana	4		*a8, Dr2, ra*
Dana	5		*aLo, Dr2, ra*
Decatur	1	USA	*Ch*
Decatur	2		*Ch*
Decatur	3		*Ch*
Decatur	4		*Ch*
Decatur	5		*Ch*
Dover	1	CAN	*a3*
Dover	2		*a3*
Dover	3		*a3*
Dover	4		*a3*

Dover	5		*none*
Drop	1	FRA	*aLo, Dr2, ra*
Drop	2		*aLo, Dr2, ra*
Drop	3		*aLo, Dr2, ra*
Drop	4		*a8*
Drop	5		*aLo, Dr2, ra*
Duet	1	GBR	*a6, Dt6, g, h*
Duet	2		*a6, Dt6, g, h*
Duet	3		*a6, Dt6, g, h*
Duet	4		*a6, Dt6, g, h*
Duet	5		*a6, Dt6, g, h*
Eckendorfer Glatta	1	DEU	*a8*
Eckendorfer Glatta	2		*a8, h*
Eckendorfer Glatta	3		*aLo*
Eckendorfer Glatta	4		*aLo*
Eckendorfer Glatta	5		*a8*
Eckendorfer Mammuth II	1	DEU	*a8, Dr2*
Eckendorfer Mammuth II	2		*Ch, Dr2*
Eckendorfer Mammuth II	3		*a8, Dr2*
Eckendorfer Mammuth II	4		*a8, Dr2*
Eckendorfer Mammuth II	5		*Ch, Dr2*
Eckendorfer Vulkan	1	DEU	*u*
Eckendorfer Vulkan	2		*u*
Eckendorfer Vulkan	3		*u*
Eckendorfer Vulkan	4		*u*
Eckendorfer Vulkan	5		*u*
Engelens Dea	1	DEU	*ra*
Engelens Dea	2		*a8, h, ra*
Engelens Dea	3		*ra*
Engelens Dea	4		*Dr2, ra*
Engelens Dea	5		*ra*
Erfa	1	DDR	*aLo, Lu*
Erfa	2		*aLo, Lu*
Erfa	3		*aLo, Lu*
Erfa	4		*aLo, Lu*
Erfa	5		*aLo, Lu*
Esther	1	DEU	*a8, Dr2, ra*
Esther	2		*a8, Dr2, ra*
Esther	3		*a8, Dr2, ra*
Esther	4		*a8, Dr2, ra*
Esther	5		*a6, ra*
Fimbull II	1	SWE	*Ch*
Fimbull II	2		*Ch*
Fimbull II	3		*Ch*
Fimbull II	4		*Ch*
Fimbull II	5		*Ch*
Firlbecks Astrid	1	DEU	*h, ra*
Firlbecks Astrid	2		*a8, h, Ln, ra*
Firlbecks Astrid	3		*h, ra*

Firlbecks Astrid	4		*h, ra*
Firlbecks Astrid	5		*a8, h, ra*
Franger	1	USA	*aLo, Lu, ra*
Franger	2		*aLo, Lu*
Franger	3		*aLo, Lu*
Franger	4		*aLo, Lu*
Franger	5		*aLo, Lu*
Freya	1	DEU	*a6*
Freya	2		*a6*
Freya	3		*a6*
Freya	4		*a6*
Freya	5		*a6*
Friedrichswerther Berg	1	DEU	*aLo, Dr2*
Friedrichswerther Berg	2		*aLo, Dr2*
Friedrichswerther Berg	3		*aLo, Dr2*
Friedrichswerther Berg	4		*aLo, Dr2*
Friedrichswerther Berg	5		*aLo, Dr2*
Frolic	1	GBR	*g*
Frolic	2		*g*
Frolic	3		*g*
Frolic	4		*g*
Frolic	5		*g*
Frost	1	SWE	*a6, h*
Frost	2		*a6, h*
Frost	3		*a6, h*
Frost	4		*a6, h*
Frost	5		*a6, h*
Gerum	1	BGR	*a13*
Gerum	2		*a13*
Gerum	3		*a13*
Gerum	4		*a13*
Gerum	5		*a13*
GK Eszter	1	HUN	*a6, h, ra*
GK Eszter	2		*a6, h, ra*
GK Eszter	3		*a6, h, ra*
GK Eszter	4		*a6, h, ra*
GK Eszter	5		*a6, h, ra*
GK Metal	1	HUN	*g, Ln*
GK Metal	2		*g, Ln*
GK Metal	3		*g, Ln*
GK Metal	4		*g, Ln*
GK Metal	5		*g, Ln*
Gloria	1	ROU	*a8*
Gloria	2		*a8*
Gloria	3		*a8*
Gloria	4		*a8*
Gloria	5		*a8*
Groninger	1	DEU	*aLo*
Groninger	2		*aLo*

Groninger	3		*Ch*
Groninger	4		*Ch*
Groninger	5		*aLo*
Grosier	1	GBR	*a12*
Grosier	2		*a12*
Grosier	3		*a12*
Grosier	4		*a12*
Grosier	5		*a12*
Guadiana	1	ESP	*aLo, Dr2, ra*
Guadiana	2		*Ch*
Guadiana	3		*Ch*
Guadiana	4		*Ch*
Guadiana	5		*Ch*
Hardy	1	AUT	*a8, ra*
Hardy	2		*Ch, ra*
Hardy	3		*Ch, Dr2, ra*
Hardy	4		*Dr2, ra*
Hardy	5		*ra*
Hatif de Grignon	1	FRA	*aLo*
Hatif de Grignon	2		*aLo, Dr2*
Hatif de Grignon	3		*aLo*
Hatif de Grignon	4		*aLo, Dr2*
Hatif de Grignon	5		*aLo, Dr2*
Hatvani 377	1	HUN	*Ch*
Hatvani 377	2		*Ch*
Hatvani 377	3		*Ch*
Hatvani 377	4		*a8*
Hatvani 377	5		*Ch*
Hauters Wintergerste	1	DEU	*a8, h, ra*
Hauters Wintergerste	2		*aLo*
Hauters Wintergerste	3		*Ch, Dr2, ra*
Hauters Wintergerste	4		*Ch, Dr2, ra*
Hauters Wintergerste	5		*ra*
Hokkaidou Hadaka	1	JPN	*Lu, Ru2*
Hokkaidou Hadaka	2		*Lu, Ru2*
Hokkaidou Hadaka	3		*Lu, Ru2*
Hokkaidou Hadaka	4		*Lu, Ru2*
Hokkaidou Hadaka	5		*Lu, Ru2*
Hooded 10	1	USA	*none*
Hooded 10	2		*Ch*
Hooded 10	3		*Ch*
Hooded 10	4		*Ch, ra*
Hooded 10	5		*e*
Chordzay 18	1	TJK	*a8*
Chordzay 18	2		*a8*
Chordzay 18	3		*a8*
Chordzay 18	4		*a8*
Chordzay 18	5		*a8*
Ibiza	1	BEL	*Ch, Dr2, ra, VIR*

Ibiza	2		*Ch, Lu, ra*
Ibiza	3		*Ch, Lu, ra*
Ibiza	4		*Ch, ra, VIR*
Ibiza	5		*Dr2, ra, VIR*
Intensiv 2	1	ROU	*a8, h, ra*
Intensiv 2	2		*a8, h, ra*
Intensiv 2	3		*a8, h, ra*
Intensiv 2	4		*a8, h, ra*
Intensiv 2	5		*a8, h, ra*
Iskra	1	SUN	*Ch, Dr2, ra*
Iskra	2		*Ch, Dr2, ra*
Iskra	3		*a8, Dr2, ra*
Iskra	4		*a8, Dr2, ra*
Iskra	5		*a8, Dr2, ra*
Jolante	1	DEU	*a6*
Jolante	2		*a6*
Jolante	3		*a6*
Jolante	4		*a6*
Jolante	5		*a6*
Jubilej 100	1	BGR	*Ch*
Jubilej 100	2		*Ch*
Jubilej 100	3		*Ch*
Jubilej 100	4		*Ch*
Jubilej 100	5		*a8*
Jubilejnij	1	UKR	*u*
Jubilejnij	2		*u*
Jubilejnij	3		*u*
Jubilejnij	4		*u*
Jubilejnij	5		*u*
Juduraki	1	JPN	*a8*
Juduraki	2		*a3*
Juduraki	3		*a3*
Juduraki	4		*Ch*
Juduraki	5		*Ch*
Jura	1	DEU	*a7*
Jura	2		*a7*
Jura	3		*a7*
Jura	4		*a7*
Jura	5		*a7*
Jutta	1	DDR	*a8*
Jutta	2		*a8*
Jutta	3		*a8*
Jutta	4		*a8*
Jutta	5		*a8*
Kamil	1	CSK	*aLo, Lu, Ru2*
Kamil	2		*aLo, Lu, Ru2*
Kamil	3		*aLo, Lu, Ru2*
Kamil	4		*aLo, Lu, Ru2*
Kamil	5		*aLo, Lu, Ru2*

Karcagi 1039	1	HUN	*Ch*
Karcagi 1039	2		*Ch*
Karcagi 1039	3		*Ch*
Karcagi 1039	4		*Ch*
Karcagi 1039	5		*Ch*
Karnobat	1	BGR	*a13*
Karnobat	2		*a13*
Karnobat	3		*a12, g*
Karnobat	4		*g*
Karnobat	5		*a12, g*
KIM M3 53/54 (nsl. Ruzyne)	1	CSK	*aLo*
KIM M3 53/54 (nsl. Ruzyne)	2		*aLo*
KIM M3 53/54 (nsl. Ruzyne)	3		*aLo*
KIM M3 53/54 (nsl. Ruzyne)	4		*aLo*
KIM M3 53/54 (nsl. Ruzyne)	5		*aLo*
Kirgizskij 247	1	KGZ	*aLo*
Kirgizskij 247	2		*aLo*
Kirgizskij 247	3		*aLo*
Kirgizskij 247	4		*aLo*
Kirgizskij 247	5		*aLo*
Kleinwanzlebener Record	1	DDR	*Ch*
Kleinwanzlebener Record	2		*Ch, ra*
Kleinwanzlebener Record	3		*Ch*
Kleinwanzlebener Record	4		*Ch*
Kleinwanzlebener Record	5		*Ch, ra*
Kompolti 4	1	HUN	*Ru2*
Kompolti 4	2		*Ru2*
Kompolti 4	3		*Ru2*
Kompolti 4	4		*Ru2*
Kompolti 4	5		*Ru2*
Konjicski	1	SUN	*aLo*
Konjicski	2		*a3*
Konjicski	3		*aLo*
Konjicski	4		*a8*
Konjicski	5		*aLo*
Kostek	1	POL	*aLo*
Kostek	2		*aLo*
Kostek	3		*a8*
Kostek	4		*aLo*
Kostek	5		*aLo*
Krakowski	1	POL	*none*
Krakowski	2		*none*
Krakowski	3		*none*
Krakowski	4		*none*
Krakowski	5		*none*
Krasnodarskij 2929	1	SUN	*none*
Krasnodarskij 2929	2		*none*
Krasnodarskij 2929	3		*none*
Krasnodarskij 2929	4		*none*

Krasnodarskij 2929	5		*none*
Kromir	1	CSK	*aLo, h, Lu, ra*
Kromir	2		*aLo, h, Lu, ra*
Kromir	3		*aLo, h, Lu, ra*
Kromir	4		*aLo, h, Lu, ra*
Kromir	5		*aLo, h, Lu, ra*
Kromoz	1	CSK	*aLo, h, Lu, ra*
Kromoz	2		*aLo, h, ra*
Kromoz	3		*a8, Lu, ra*
Kromoz	4		*a8, h, Ln, ra*
Kromoz	5		*aLo, h, ra*
Kruglik 21	1	SUN	*Ch*
Kruglik 21	2		*Ch*
Kruglik 21	3		*Ch*
Kruglik 21	4		*Ch*
Kruglik 21	5		*Ch*
Krusevacki	1	YUG	*none*
Krusevacki	2		*none*
Krusevacki	3		*none*
Krusevacki	4		*none*
Krusevacki	5		*none*
Kujawiak III	1	POL	*aLo*
Kujawiak III	2		*aLo*
Kujawiak III	3		*aLo, h*
Kujawiak III	4		*aLo*
Kujawiak III	5		*aLo*
Ledeci Beta	1	HUN	*Ch*
Ledeci Beta	2		*Ch*
Ledeci Beta	3		*a8*
Ledeci Beta	4		*a8*
Ledeci Beta	5		*Ch*
Leon	1	NLD	*aLo, Dr2, ra*
Leon	2		*none*
Leon	3		*a8*
Leon	4		*aLo, Dr2, ra*
Leon	5		*aLo, Dr2, ra*
Local (Balkan)	1	GRC	*a8, ra*
Local (Balkan)	2		*a8*
Local (Balkan)	3		*u*
Local (Balkan)	4		*aLo*
Local (Balkan)	5		*aLo*
Local (Merkez-Kaza)	1	TUR	*at, h*
Local (Merkez-Kaza)	2		*at, h*
Local (Merkez-Kaza)	3		*at, h*
Local (Merkez-Kaza)	4		*at, h*
Local (Merkez-Kaza)	5		*at, h*
Lomerit	1	DEU	*aLo, VIR*
Lomerit	2		*aLo, VIR*
Lomerit	3		*aLo, VIR*

Lomerit	4		*aLo, VIR*
Lomerit	5		*aLo, VIR*
Lunet	1	CSK	*aLo, Lu, Ru2*
Lunet	2		*aLo, Lu, Ru2*
Lunet	3		*aLo, Lu, Ru2*
Lunet	4		*aLo, Lu, Ru2*
Lunet	5		*aLo, Lu, Ru2*
Luran	1	CZE	*aLo, Lu, ra, Ru2*
Luran	2		*aLo, Lu, ra, Ru2*
Luran	3		*aLo, Lu, ra, Ru2*
Luran	4		*aLo, Lu, ra, Ru2*
Luran	5		*aLo, Lu, ra, Ru2*
Luxor	1	CSK	*aLo, h, Lu, ra, Ru2*
Luxor	2		*aLo, h, Lu, ra, Ru2*
Luxor	3		*aLo, h, Lu, ra, Ru2*
Luxor	4		*aLo, h, Lu, ra, Ru2*
Luxor	5		*aLo, h, Lu, ra, Ru2*
Maguelone	1	FRA	*aLo, Dr2*
Maguelone	2		*aLo*
Maguelone	3		*a8, ra*
Maguelone	4		*aLo*
Maguelone	5		*a8, Dr2, ra*
Marconee	1	USA	*e*
Marconee	2		*e*
Marconee	3		*e*
Marconee	4		*e*
Marconee	5		*e*
Marinka	1	NLD	*a7*
Marinka	2		*a7*
Marinka	3		*a7*
Marinka	4		*a7*
Marinka	5		*a7*
Marjorie	1	FRA	*a8, h*
Marjorie	2		*a8, h*
Marjorie	3		*a8, h*
Marjorie	4		*a8, h*
Marjorie	5		*a8, h*
Marna	1	FRA	*ra*
Marna	2		*ra*
Marna	3		*ra*
Marna	4		*ra*
Marna	5		*ra*
Martha	1	AUT	*g*
Martha	2		*g*
Martha	3		*g*
Martha	4		*g*
Martha	5		*g*
Mc Nair 601	1	USA	*e*
Mc Nair 601	2		*e*

Mc Nair 601	3		*e*
Mc Nair 601	4		*e*
Mc Nair 601	5		*e*
Merlot	1	DEU	*a6, h, ra*
Merlot	2		*a6, h, ra*
Merlot	3		*a6, h, ra*
Merlot	4		*a6, h, ra*
Merlot	5		*a6, h, ra*
Michigan Winter	1	USA	*aLo*
Michigan Winter	2		*aLo*
Michigan Winter	3		*aLo*
Michigan Winter	4		*aLo*
Michigan Winter	5		*aLo*
Mijana	1	MDA	*aLo*
Mijana	2		*aLo*
Mijana	3		*aLo*
Mijana	4		*aLo*
Mijana	5		*aLo*
Miraj 1	1	ROU	*Ch, h, ra*
Miraj 1	2		*Ch, ra, h, VIR*
Miraj 1	3		*Ch, ra*
Miraj 1	4		*Ch, ra*
Miraj 1	5		*Ch, h, ra*
Mironovskij 82	1	UKR	*a12, aLo, g, Lu*
Mironovskij 82	2		*a12, aLo, g, Lu*
Mironovskij 82	3		*a12, aLo, g, Lu*
Mironovskij 82	4		*aLo, Lu, Ru2*
Mironovskij 82	5		*a12, aLo, Lu*
Monaco	1	FRA	*ra*
Monaco	2		*ra*
Monaco	3		*ra*
Monaco	4		*ra*
Monaco	5		*ra*
Muellers Boehmerwaelder	1	DEU	*aLo*
Muellers Boehmerwaelder	2		*aLo*
Muellers Boehmerwaelder	3		*Ch, Dr2, ra*
Muellers Boehmerwaelder	4		*aLo*
Muellers Boehmerwaelder	5		*Ch, Dr2, ra*
Nachicivandany	1	AZE	*Ru2*
Nachicivandany	2		*Ru2*
Nachicivandany	3		*Ru2*
Nachicivandany	4		*Ru2*
Nachicivandany	5		*Ru2*
Nakaizumi Zairai	1	JPN	*none*
Nakaizumi Zairai	2		*none*
Nakaizumi Zairai	3		*none*
Nakaizumi Zairai	4		*none*
Nakaizumi Zairai	5		*a8*
Nelly	1	DEU	*a13*

Nelly	2		*a13*
Nelly	3		*a13*
Nelly	4		*a13*
Nelly	5		*a13*
Noveta	1	DNK	*a8, h, Lu, Ru2*
Noveta	2		*a8, h, Lu, Ru2*
Noveta	3		*a8, h, Lu, Ru2*
Noveta	4		*a8, h, Lu, Ru2*
Noveta	5		*a8, h, Lu, Ru2*
Novosadski 703	1	YUG	*a8*
Novosadski 703	2		*a8*
Novosadski 703	3		*a8*
Novosadski 703	4		*aLo, Lu, Ru2*
Novosadski 703	5		*aLo, Lu, Ru2*
O.A.C. Halton	1	CAN	*a8*
O.A.C. Halton	2		*a8*
O.A.C. Halton	3		*a8*
O.A.C. Halton	4		*a8*
O.A.C. Halton	5		*a8*
Odesskij 2095	1	UKR	*a6, h*
Odesskij 2095	2		*a6, h, ra*
Odesskij 2095	3		*a6, h, ra*
Odesskij 2095	4		*a6, h, ra*
Odesskij 2095	5		*a6, h, ra*
Okal	1	CSK	*aLo, h, Lu, ra, Ru2*
Okal	2		*aLo, h, Lu, ra, Ru2*
Okal	3		*aLo, h, Lu, ra, Ru2*
Okal	4		*aLo, h, Lu, ra, Ru2*
Okal	5		*aLo, h, Lu, ra, Ru2*
Okayama mitsuki Hadaka	1	JPN	*u*
Okayama mitsuki Hadaka	2		*u*
Okayama mitsuki Hadaka	3		*u*
Okayama mitsuki Hadaka	4		*u*
Okayama mitsuki Hadaka	5		*u*
Oksamyt	1	UKR	*aLo*
Oksamyt	2		*aLo*
Oksamyt	3		*aLo*
Oksamyt	4		*aLo*
Oksamyt	5		*aLo*
Oma	1	USA	*a8*
Oma	2		*a8*
Oma	3		*a8*
Oma	4		*a8, Ru2*
Oma	5		*aLo, Ru2*
Opolski 152	1	POL	*none*
Opolski 152	2		*none*
Opolski 152	3		*none*
Opolski 152	4		*none*
Opolski 152	5		*none*

Pallidum 310/1	1	AZE	*u*
Pallidum 310/1	2		*u*
Pallidum 310/1	3		*u*
Pallidum 310/1	4		*u*
Pallidum 310/1	5		*u*
Pallidum 728/15	1	SUN	*aLo*
Pallidum 728/15	2		*aLo*
Pallidum 728/15	3		*a8*
Pallidum 728/15	4		*a8, Dr2, ra*
Pallidum 728/15	5		*aLo*
Pamina	1	DDR	*aLo, Lu*
Pamina	2		*aLo, Lu*
Pamina	3		*a6, ra*
Pamina	4		*aLo, Lu*
Pamina	5		*aLo, Lu*
Pavlovicky	1	CSK	*a8*
Pavlovicky	2		*a8*
Pavlovicky	3		*a8*
Pavlovicky	4		*a8*
Pavlovicky	5		*a8*
Peragis Mittelfruehe	1	DEU	*a8*
Peragis Mittelfruehe	2		*a8*
Peragis Mittelfruehe	3		*a8*
Peragis Mittelfruehe	4		*a8*
Peragis Mittelfruehe	5		*a8*
Perga	1	DEU	*h, ra*
Perga	2		*h, ra*
Perga	3		*h, ra*
Perga	4		*Ch, h, ra*
Perga	5		*Ch, h, ra*
Persikum 64	1	SUN	*h*
Persikum 64	2		*h*
Persikum 64	3		*u*
Persikum 64	4		*u*
Persikum 64	5		*u*
Poljarnyj 14	1	SUN	*Ch, Dr2, ra*
Poljarnyj 14	2		*Ch, Dr2, ra*
Poljarnyj 14	3		*Ch, Dr2, ra*
Poljarnyj 14	4		*Ch, Dr2, ra*
Poljarnyj 14	5		*none*
Po-ri	1	PRK	*h*
Po-ri	2		*h*
Po-ri	3		*h*
Po-ri	4		*a8*
Po-ri	5		*Dr2, ra*
Probsdorfer Robusta	1	AUT	*aLo, Dr2*
Probsdorfer Robusta	2		*Ch, h, ra*
Probsdorfer Robusta	3		*aLo, Dr2*
Probsdorfer Robusta	4		*h*

Probsdorfer Robusta	5		*aLo*
Protidor	1	ITA	*a12, g, VIR*
Protidor	2		*a12, g, VIR*
Protidor	3		*g*
Protidor	4		*a12, g, VIR*
Protidor	5		*g*
Ragusa 34-40	1	YUG	*a8, h, ra*
Ragusa 34-40	2		*Ch, Dr2, ra*
Ragusa 34-40	3		*a8, h*
Ragusa 34-40	4		*a8*
Ragusa 34-40	5		*Ch, h, ra*
Rapidan	1	USA	*a8, VIR*
Rapidan	2		*a8, Ru3*
Rapidan	3		*a8, VIR*
Rapidan	4		*a8, Ru4*
Rapidan	5		*a8, VIR*
Rengapolbori	1	PRK	*Ch, Dr2, ra, VIR*
Rengapolbori	2		*Ch, Dr2, ra, VIR*
Rengapolbori	3		*Ch, Dr2, ra, VIR*
Rengapolbori	4		*Ch, Dr2, ra, VIR*
Rengapolbori	5		*a8, Dr2, ra, VIR*
Reni	1	DEU	*ra*
Reni	2		*ra*
Reni	3		*ra*
Reni	4		*ra*
Reni	5		*Ch, ra*
Rozen	1	BGR	*a6, h*
Rozen	2		*a8, h, ra*
Rozen	3		*a6, h*
Rozen	4		*a8, h*
Rozen	5		*a6, h*
Russe 85	1	BGR	*h*
Russe 85	2		*h*
Russe 85	3		*h*
Russe 85	4		*h*
Russe 85	5		*h*
Scorpio	1	BEL	*aLo, g*
Scorpio	2		*aLo, g*
Scorpio	3		*aLo, g*
Scorpio	4		*aLo, g*
Scorpio	5		*aLo, g*
Schwarze Wintergerste	1	DEU	*aLo*
Schwarze Wintergerste	2		*aLo*
Schwarze Wintergerste	3		*aLo*
Schwarze Wintergerste	4		*aLo*
Schwarze Wintergerste	5		*aLo*
Sigra	1	DEU	*Dr2, ra*
Sigra	2		*Dr2, ra*
Sigra	3		*Dr2, ra*

Sigra	4		*Dr2, ra*
Sigra	5		*Dr2, ra*
Silke	1	DEU	*a6, ra*
Silke	2		*a6, ra*
Silke	3		*a6, ra*
Silke	4		*a6, ra*
Silke	5		*a6, ra*
Sirvandany 30	1	AZE	*aLo*
Sirvandany 30	2		*none*
Sirvandany 30	3		*aLo*
Sirvandany 30	4		*aLo*
Sirvandany 30	5		*none*
Slaski II	1	POL	*aLo*
Slaski II	2		*aLo*
Slaski II	3		*aLo*
Slaski II	4		*aLo*
Slaski II	5		*aLo*
Sorna	1	DDR	*a13*
Sorna	2		*a13*
Sorna	3		*aLo, Lu, ra*
Sorna	4		*a13*
Sorna	5		*a13*
Strengs Dura	1	DEU	*Ch, Dr2, ra*
Strengs Dura	2		*a8, Lu, ra*
Strengs Dura	3		*Ch, ra*
Strengs Dura	4		*Ch, Dr2, ra*
Strengs Dura	5		*Ch, ra*
Stupicky dvourady	1	CSK	*none*
Stupicky dvourady	2		*none*
Stupicky dvourady	3		*none*
Stupicky dvourady	4		*none*
Stupicky dvourady	5		*u*
Stupicky sestirady	1	CSK	*Ch, Ru2*
Stupicky sestirady	2		*aLo*
Stupicky sestirady	3		*aLo*
Stupicky sestirady	4		*aLo*
Stupicky sestirady	5		*aLo*
Sumavsky	1	CSK	*aLo*
Sumavsky	2		*aLo*
Sumavsky	3		*aLo*
Sumavsky	4		*aLo*
Sumavsky	5		*none*
Tamaris	1	FRA	*h, ra*
Tamaris	2		*h, ra*
Tamaris	3		*h, ra*
Tamaris	4		*h, ra*
Tamaris	5		*h, ra*
Tiffany	1	DEU	*a7*
Tiffany	2		*a7*

Tiffany	3		*a7*
Tiffany	4		*a7*
Tiffany	5		*a7*
Traminer	1	DEU	*IM9, St*
Traminer	2		*IM9, St*
Traminer	3		*IM9, St*
Traminer	4		*IM9, St*
Traminer	5		*IM9, St*
Tschermaks Vierzeilige Glatte	1	AUT	*a8*
Tschermaks Vierzeilige Glatte	2		*a8*
Tschermaks Vierzeilige Glatte	3		*a8*
Tschermaks Vierzeilige Glatte	4		*a8*
Tschermaks Vierzeilige Glatte	5		*a8*
Uzen-czjan 64	1	TKM	*aLo, Lu*
Uzen-czjan 64	2		*aLo*
Uzen-czjan 64	3		*none*
Uzen-czjan 64	4		*Lu, Ru2*
Uzen-czjan 64	5		*a8*
Ventitre	1	ITA	*aLo, ra, Ru2*
Ventitre	2		*aLo, ra, Ru2*
Ventitre	3		*a8*
Ventitre	4		*a8*
Ventitre	5		*a8*
Vilna	1	NLD	*La, ra*
Vilna	2		*La, ra*
Vilna	3		*La, ra*
Vilna	4		*La, ra*
Vilna	5		*La, ra*
VIR 6139	1	ARM	*VIR*
VIR 6139	2		*VIR*
VIR 6139	3		*VIR*
VIR 6139	4		*VIR*
VIR 6139	5		*VIR*
Virgo	1	NLD	*La, ra*
Virgo	2		*a8, h*
Virgo	3		*La, ra*
Virgo	4		*La, ra*
Virgo	5		*La, ra*
Vogelsanger Gold	1	DEU	*a8*
Vogelsanger Gold	2		*a8*
Vogelsanger Gold	3		*a8*
Vogelsanger Gold	4		*a8*
Vogelsanger Gold	5		*a8*
Volbar	1	USA	*Ch, Dr2, ra*
Volbar	2		*Ch, Dr2, ra*
Volbar	3		*Ch, Dr2, ra*
Volbar	4		*Ch, Dr2, ra*
Volbar	5		*Dr2, ra*
Wade	1	USA	*Ch, Dr2, Lu, Ru2*

Wade	2		*Ch, Dr2, Lu, Ru2*
Wade	3		*Ch, Dr2, Lu, Ru2*
Wade	4		*Ch, Dr2, Lu, Ru2*
Wade	5		*Ch, Dr2, Lu, Ru2*
Will	1	USA	*a7*
Will	2		*a7*
Will	3		*a7*
Will	4		*a7, h*
Will	5		*a7, h*
Wong	1	USA	*Wo*
Wong	2		*Wo*
Wong	3		*Wo*
Wong	4		*Wo*
Wong	5		*Wo*
Zalarinec	1	SUN	*none*
Zalarinec	2		*aLo, h*
Zalarinec	3		*none*
Zalarinec	4		*none*
Zalarinec	5		*none*
Zend	1	TUR	*a8*
Zend	2		*a8*
Zend	3		*a8*
Zend	4		*a8*
Zend	5		*a8*
Zenit	1	BGR	*a13*
Zenit	2		*a13*
Zenit	3		*a13*
Zenit	4		*a13*
Zenit	5		*a13*

[1] ARM Armenia, AUT Austria, AZE Azerbaijan, BEL Belgium, BGR Bulgaria, CAN Canada, CSK Czechoslovakia, CZE Czech Republic, DDR German Democratic Republic, DEU Germany, DNK Denmark, DZA Algeria, ESP Espana, ETH Ethiopia, FRA France, GBR Great Britain, GRC Greece, HUN Hungary, CHE Switzerland, ITA Italy, JPN Japan, KGZ Kyrgyzstan, MDA Moldova, NLD Netherlands, POL Poland, PRK Democratic People's Republic of Korea, ROU Romania, SUN Soviet Union, SWE Sweden, TJK Tajikistan, TKM Turkmenistan, TUR Turkey, UKR Ukraine, USA United States of America, YUG Yugoslavia.
